# Effect of Oral Nirmatrelvir on Long COVID Symptoms: 4 Cases and Rationale for Systematic Studies

**DOI:** 10.20411/pai.v7i1.518

**Published:** 2022-06-24

**Authors:** Michael J. Peluso, Khamal Anglin, Matthew S. Durstenfeld, Jeffrey N. Martin, J. Daniel Kelly, Priscilla Y. Hsue, Timothy J. Henrich, Steven G. Deeks

**Affiliations:** 1 Division of HIV, Infectious Diseases, and Global Medicine, University of California, San Francisco, San Francisco, CA; 2 Department of Epidemiology and Biostatistics, University of California, San Francisco, San Francisco, CA; 3 Division of Cardiology, University of California, San Francisco, San Francisco, CA; 4 Division of Experimental Medicine, University of California, San Francisco, San Francisco, CA

**Keywords:** SARS-CoV-2, Long COVID, post-acute sequelae of SARS-CoV-2 (PASC), antiviral therapy, nirmatrelvir, Paxlovid

## Abstract

**Background:**

Efforts to understand the impact of SARS-CoV-2 variants, vaccine status, and treatment on the development and persistence of Long COVID have intensified.

**Methods:**

We report 4 sequential cases from a post-COVID cohort study demonstrating variability in outcomes following differentially timed nirmatrelvir therapy, received as part of clinical care.

**Results:**

In the first case, the participant experienced symptomatic rebound and developed Long COVID despite early initiation of antiviral therapy. In the next 2 cases, participants reported improvement in persistent COVID symptoms when nirmatrelvir was taken 25 and 60 days following initial symptom onset. In the final case, an individual with presumed Long COVID for 2 years reported substantial improvement in chronic symptoms when taking nirmatrelvir following SARS-CoV-2 re-infection.

**Conclusions:**

These anecdotes suggest that systematic study of antiviral therapy for Long COVID is warranted.

## TO THE EDITOR

Many individuals do not fully recover from acute SARS-CoV-2 infection (“Long COVID,” a type of post-acute sequelae of SARS-CoV-2[(PASC]). Efforts to prevent or reverse this potentially disabling syndrome are just now emerging. Here, we report 4 cases in which individuals were treated with nirmatrelvir/ritonavir at various stages following infection. We believe these cases provide rationale for studying antiviral therapies in people with Long COVID.

Participants were sequential volunteers in the UCSF Long-term Impact of Infection with Novel Coronavirus (LIINC) study (NCT04362150), who enrolled because of a history of Long-COVID symptoms and reported a history of nirmatrelvir/ritonavir use. The study was approved by the UCSF Institutional Review Board. Volunteers provided written informed consent prior to collection of clinical data and consented to the presentation of their cases.

### Case 1

**Figure 1.**
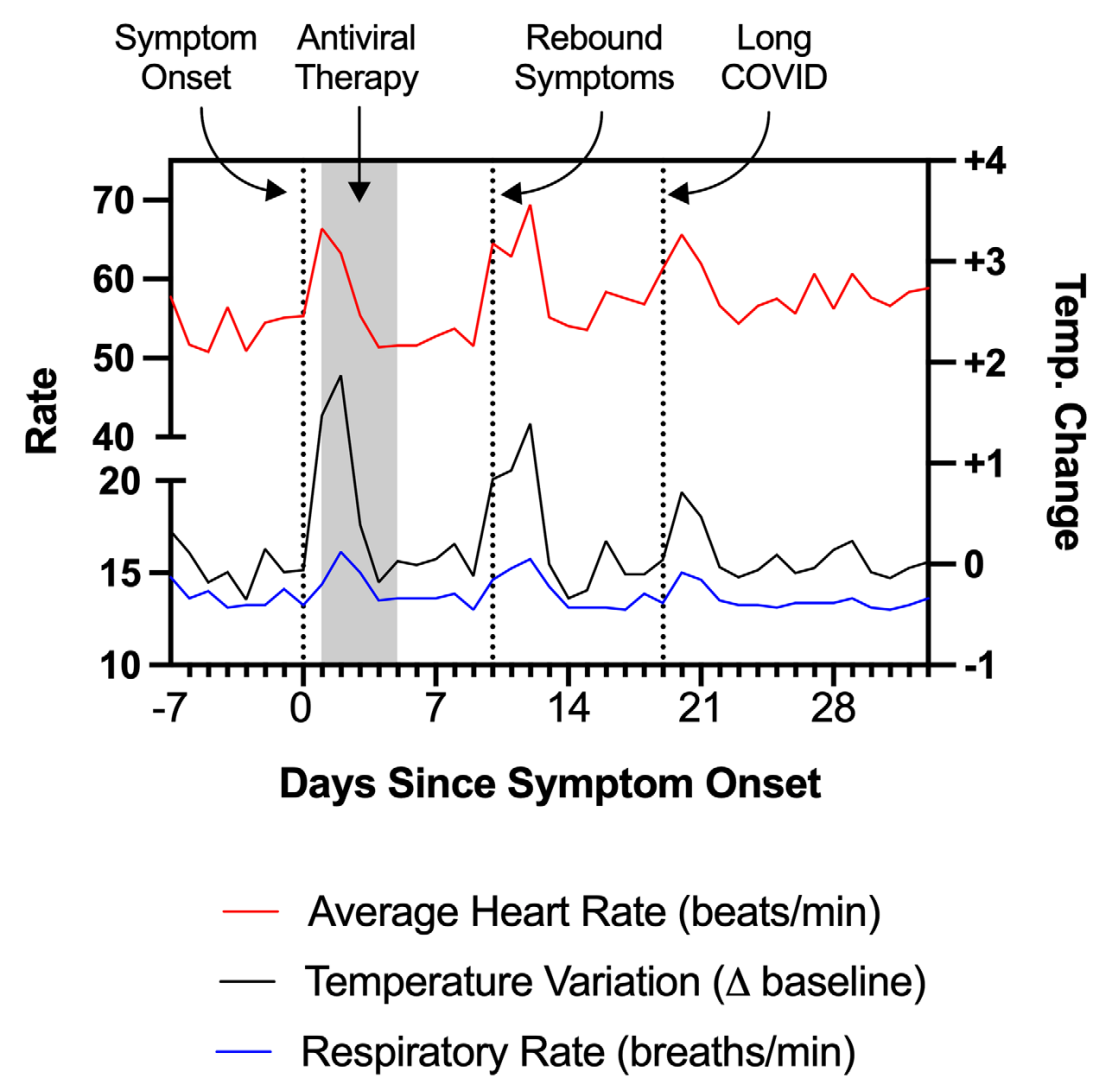
Physiologic measurements recorded by a personal fitness device used by Patient 1, showing rebound tachycardia, tachypnea, and elevated body temperature coinciding with completion of antiviral therapy. The individual subsequently developed additional symptoms which have persisted, consistent with Long COVID.

A 48-year-old man with a past medical history of presumed Bechet's disease and taking colchicine developed fever, worsening headache, and pharyngitis in Spring 2022. He had previously received 2 doses of the mRNA-1273 SARS-CoV-2 vaccine and 1 dose of the BNT162b2 vaccine; the most recent was 5 months prior. A rapid antigen test was positive, as was a confirmatory PCR test. He was prescribed a 5-day course of nirmatrelvir/ritonavir, which he initiated within 24 hours of symptom onset, and experienced rapid improvement in his systemic symptoms. However, approximately 4 days following completion of the 5-day course, he experienced rebound symptoms with recurrence of fever, fatigue, rhinorrhea, cough, chest pain, rash on his upper and lower extremities, and trouble concentrating (“brain fog”). During this period, he wore a personal fitness device which recorded certain physiologic measurements including heart rate, respiratory rate, and change from baseline body temperature (Figure 1). Approximately 3 weeks following the positive test and despite prior antiviral therapy, he experienced worsening of his fatigue and associated chest soreness, palpitations, brain fog, and symptoms of post-exertional malaise, which persisted beyond 30 days following initial symptom onset. At 6 weeks, he had a normal echocardiogram including normal left ventricular ejection fraction of 69%, normal right and left ventricular strain, and normal diastolic function. On orthostatic testing he had dizziness but no evidence of ortho-static hypotension or tachycardia. On maximal symptom-limited cardiopulmonary exercise testing, he had very reduced peak VO_2_ (12.4ml/kg/min, 60% predicted) and reduced maximum heart rate (79% predicted, adjusted heart rate reserve 64%) consistent with chronotropic incompetence.

### Case 2

A 42-year-old man with no significant medical history developed rhinorrhea and pharyngitis in early 2022, followed by fatigue, myalgia, and a pruritic rash on his upper extremities and groin that persisted for approximately 10 days. He had previously received 3 doses of the BNT162b2 SARS-CoV-2 vaccine; the most recent was 2 months prior. A SARS-CoV-2 antigen test was positive when performed 2 days after symptom onset; multiple additional antigen tests were subsequently positive. On Day 11, an antigen test was negative, and all of his symptoms resolved. Approximately 2 weeks later, he experienced new onset of severe myalgia and bone pain across his upper body, which he described as post-exertional soreness in the absence of exertion. There was an associated increase in fatigue, as well as heightened awareness of breathing (described as “lung soreness”). These symptoms persisted, and he contacted his primary care doctor for evaluation approximately 10 days later. At this visit, he noted that he was experiencing ongoing fatigue, and described being at 80% of his pre-COVID baseline health. Laboratory testing was notable only for vitamin D insufficiency (25 ng/mL); a chest X-ray showed no abnormalities.

Approximately 7 weeks following initial symptom onset, his symptoms worsened. He experienced ongoing myalgia, severe fatigue, post-exertional malaise, and trouble concentrating (“brain fog”). These symptoms profoundly affected his ability to perform his activities of daily living, and he felt substantially debilitated, reporting that he was at 40%-50% of his pre-COVID baseline health, and that he was spending the majority of the day resting. He began to seek care for Long COVID because he was concerned about the duration of his symptoms. As his symptoms continued to worsen, he was re-exposed to SARS-CoV-2 when his spouse and children tested positive on antigen tests. A repeat antigen test was negative, but he experienced further worsening of his symptoms which his provider attributed to possible re-infection with SARS-CoV-2. In this context, he received a prescription for nirmatrelvir/ritonavir.

Within days of re-exposure, the patient began to note an improvement in his persistent symptoms, while his family members continued to experience worsening symptoms. After symptomatic improvement for 1-2 days, he initiated a 5-day course of nirmatrelvir/ritonavir. During this period, he continued to experience improvement in his symptoms. While they have not resolved entirely as of 2 weeks later, he reports that he is gradually approaching his baseline health.

### Case 3

A 43-year-old woman with no significant medical history developed cough and pharyngitis in Spring 2022. She had previously received 3 doses of the BNT162b2 SARS-CoV-2 vaccine; the most recent was 4 months prior. While a PCR test was initially negative, she and one of her children subsequently tested positive on an antigen test 5 days later. She did not initially receive antiviral therapy. Over the course of the subsequent 3 weeks, she began to experience worsening fatigue and malaise, with associated myalgia and trouble concentrating (“brain fog”); 3 weeks following initial symptom onset she was spending the majority of the day resting and was unable to easily complete her activities of daily living. She received a prescription for nirmatrelvir/ritonavir, which she began 25 days following initial symptom onset. One day following completion of therapy, she experienced improvement in her fatigue symptoms. While she has residual shortness of breath and myalgias, she has now been able to re-engage with the usual activities of daily living.

### Case 4

In March 2020, a 47-year-old physician with a history of asthma developed an acute viral illness characterized by 12 days of sore throat, fatigue, diarrhea, and myalgia while working on an inpatient COVID-19 ward. She was not tested for SARS-CoV-2 infection due to the lack of availability of nucleic acid testing and the absence of fever, cough, or recent travel to China, which were strict criteria for testing during this time period. While many of her acute symptoms improved and she returned to work, she subsequently experienced progressive symptoms which included worsening myalgia, trouble concentrating (“brain fog”), chest pain, chronic diarrhea, and intermittent debilitating fatigue which persisted over the subsequent 20 months; this was presumed to represent Long COVID. She received 3 doses of the BNT162b2 SARS-CoV-2 vaccine with minimal change in her symptoms, although the third dose resulted in a week of intensified chest pain. Her Long Covid symptoms continued to progress to the point that she decided to take a leave of absence from work in early Winter 2022. Two months later, she was re-infected with SARS-CoV-2 and prescribed nirmatrelvir/ritonavir given her elevated risk for severe disease. She began treatment on Day 2 of her acute re-infection. She reported that within two days of initiating therapy, she began to experience relief from her chronic Long COVID symptoms, with substantial improvement in myalgia, diarrhea, and chest pain.

## DISCUSSION

As the SARS-CoV-2 pandemic continues to evolve, efforts to understand variability in COVID-19 recovery, as well as the impact of factors including viral variants, vaccine status, and COVID-19 treatment on the development and persistence of Long COVID symptoms have intensified. This case series demonstrates that variability in the timing of antiviral therapy as well as other potential determinants of Long COVID may be associated with different outcomes and underscores the need for systematic study of antiviral therapy for this disease condition during both the acute and convalescent stages.

It has been suggested that the viral burden during acute infection may be an important determinant of Long COVID, [1] and that early antiviral therapy might mitigate this risk. In Case 1, the individual took nirmatrelvir/ritonavir according to the recent Emergency Use Authorization (EUA) criteria [2] and shortly thereafter experienced rebound symptoms, which were associated with physiologic changes measured using a fitness device. Symptomatic relapses of SARS-CoV-2 infection are just now starting to be reported. [3] Although he was not re-tested during this period, it is possible that this coincided with viral rebound upon the completion of therapy. Concerningly, he subsequently experienced worsening post-infectious symptoms which now meet US Centers for Disease Control (CDC) criteria for Long COVID [4]. In this case, the participant already has objective findings (reduced peak VO2 and chronotropic incompetence) consistent with PASC, which may contribute to reduced exercise capacity and have been shown to persist in some cases. [5–7] This suggests that although a short course of early antiviral therapy is adequate to prevent severe acute disease in high-risk patients, [8] it may be insufficient to prevent the development of Long COVID, and those experiencing rebound symptoms could remain at risk.

A related hypothesis is that SARS-CoV-2 may persist for weeks to months in some individuals, causing inflammation, local tissue damage, and end-organ disease. [9–11] If this turns out to be the case, antiviral therapy during the acute and post-acute stages may prevent or even reverse Long COVID. Although not approved under the EUA, [2] there are reports emerging about individuals accessing oral antiviral therapy at later points in the disease course and the potential effects of these therapies. [12] We present 3 cases in which individuals were able to access nirmatrelvir/ritonavir for clinical care in the setting of persistent COVID-19 symptoms. There were notable differences between these cases, including the timing of antiviral therapy from initial infection (<30 days, >60 days, and 2 years), as well as potential lineage differences based on the timing of infection (Spring 2022, Winter 2021-2022, and March 2020). While single anecdotes must be interpreted with caution, these cases emphasize the urgent need for carefully designed studies to assess the impact of antiviral therapy beyond the acute window. In particular, the final case suggests a potential effect even in people with long-standing Long COVID symptoms. Confirmation of a benefit in the context of such studies would support the hypothesis that persistent viral activity, particularly in the tissues, [9–11, 13] could be one contributor to ongoing symptoms in Long COVID.

An additional factor in 2 of these cases was re-exposure and/or re-infection events; in one case the individual began to experience improvement in Long COVID symptoms which had until that point been escalating. Although vaccines are likely to reduce the risk of developing Long COVID, [14] there are numerous, but inconsistent, reports of the impact of SARS-CoV-2 vaccination on pre-existing Long COVID symptoms. [15, 16] A dysregulated immune response has been proposed as a potential mechanism underlying Long COVID pathophysiology. [1, 17–18] The fact that re-exposure to viral antigen may have led to symptomatic improvement is intriguing, suggesting the possibility that a dysregulated immune response, if present, could be recalibrated.

Although this case series is limited by a lack of intensive physiologic and laboratory measurements throughout the disease course, we believe these clinical anecdotes are informative as investigators try to understand the pathophysiology that drives the development and persistence of Long COVID. They suggest that antiviral therapy and/or antigen re-exposure could potentially alter the complex interplay between viral replication and the host immune response that likely underlies this syndrome but raise concern that brief early antiviral therapy alone may be insufficient to prevent the development of Long COVID.
